# Role of p85α in neutrophil extra- and intracellular reactive oxygen species generation

**DOI:** 10.18632/oncotarget.8500

**Published:** 2016-03-30

**Authors:** Xing Jun Li, Lisa Deng, Stephanie L. Brandt, Charles B. Goodwin, Peilin Ma, Zhenyun Yang, Raghu S. Mali, Ziyue Liu, Reuben Kapur, C. Henrique Serezani, Rebecca J. Chan

**Affiliations:** ^1^ Department of Pediatrics, Indianapolis, IN, USA; ^2^ Herman B Wells Center for Pediatric Research, Indianapolis, IN, USA; ^3^ Department of Medical & Molecular Genetics, Indianapolis, IN, USA; ^4^ Department of Microbiology & Immunology, Indianapolis, IN, USA; ^5^ Department of Pathology, Indiana University School of Medicine, Indianapolis, IN, USA; ^6^ Department of Biostatistics, Indiana University Richard M. Fairbanks School of Public Health, Indianapolis, IN, USA

**Keywords:** PI3K, p85α, neutrophil, NADPH oxidase, MRSA, Immunology and Microbiology Section, Immune response, Immunity

## Abstract

Drug resistance is a growing problem that necessitates new strategies to combat pathogens. Neutrophil phagocytosis and production of intracellular ROS, in particular, has been shown to cooperate with antibiotics in the killing of microbes. This study tested the hypothesis that p85α, the regulatory subunit of PI3K, regulates production of intracellular ROS. Genetic knockout of p85α in mice caused decreased expression of catalytic subunits p110α, p110β, and p110δ, but did not change expression levels of the NADPH oxidase complex subunits p67*^phox^*, p47*^phox^*, and p40*^phox^*. When p85α, p55α, and p50α (all encoded by *Pik3r1*) were deleted, there was an increase in intracellular ROS with no change in phagocytosis in response to both Fcγ receptor and complement receptor stimulation. Furthermore, the increased intracellular ROS correlated with significantly improved neutrophil killing of both methicillin-susceptible and methicillin-resistant *S. aureus*. Our findings suggest inhibition of p85α as novel approach to improving the clearance of resistant pathogens.

## INTRODUCTION

*Staphylococcus aureus* make up a large proportion of human infections worldwide, causing various diseases that range from acute skin infections to life-threatening systemic toxic shock syndromes. The rise of methicillin-resistant *S. aureus* (MRSA) and other antibiotic-resistant strains has sparked the need for new treatment strategies in both immunodeficient and immunocompetent individuals [1–5]. Neutrophils are part of the innate immune system and are critical for clearing *S. aureus* infections. They are the first responders to invading bacteria and kill microbes using reactive oxygen species (ROS) produced by the NADPH oxidase complex [1, 6–10]. During ingestion, neutrophils first form a phagosomal cup, which then becomes a fully internalized phagosome where microorganisms are isolated and exposed to toxic levels of superoxide (O_2_^−^) and other reactive oxygen species (ROS) [11]. The NADPH oxidase complex is located within the phagosome membrane and it is made up of membrane-bound gp91*^phox^* and p22*^phox^*; and cytosolic p47*^phox^*, p67*^phox^*, p40*^phox^*, and Rac2 [12, 13]. Individuals with chronic granulomatous disease (CGD) demonstrate the importance of NADPH oxidase function for human health, as these patients lack a functional NADPH oxidase complex, and thus suffer from recurrent and severe bacterial and fungal infections [10, 14, 15].

A variety of receptors on neutrophil membranes contribute to the recognition of opsonized material. Two of the most important receptors are Fcγ receptors (FcγRs), which bind to IgG-coated pathogens, and complement receptors (CRs), which bind C3b on complement-coated pathogens [16–18]. FcγR or CR binding causes the cytosolic components of NADPH oxidase to translocate to gp91*^phox^*/p22*^phox^* at the membrane. Downstream of these receptors are many signaling molecules that regulate NADPH oxidase assembly and activation, including Class IA phosphoinositide 3-kinase (PI3K). PI3K is a heterodimer consisting of a regulatory subunit (p85α, p55α, p50α, or p85β) and a catalytic subunit (p110α, p110β, or p110δ) and phosphorylates the lipid PI(4,5)P_2_ to produce PI(3,4,5)P_3_ [12, 13]. Furthermore, pharmacologic inhibition and genetic ablation of the catalytic subunits have been shown to decrease neutrophil ROS production in response to IgG-zymosan and *Aspergillus fumigatus* hyphae [16, 19]. However, the specific role of p85α, the most abundant regulatory subunit of Class IA PI3K, has not been fully studied.

We previously found that a functional binding site on p47*^phox^* for Class IA PI3K-derived phospho-lipids, PI(3,4)P_2_ and PI(3,4,5)P_3,_ is needed for extracellular ROS production, but is dispensable for intracellular ROS production during early phagocytosis [20]. This finding is consistent with the observation that PI(3,4)P_2_ and PI(3,4,5)P_3_ are found on the early phagosomal cup at the location of and during the time of extracellular ROS production, but are not detected on the mature, internalized phagosome. Notably, p85α, the regulatory subunit of Class IA PI3K and thus necessary for PI(3,4,5)P_3_ production, remains associated with the phagosome membrane even when PI(3,4,5)P_3_ is no longer present [21]. These observations led us to hypothesize that p85α differentially influences extracellular and intracellular NADPH oxidase activity and performs a function on the internalized, sealed phagosome independent of PI(3,4,5)P_3_ production.

To test this hypothesis, we used neutrophils lacking p85α, p55α, and p50α (encoded by *Pik3r1*), and distinguished production of extra- and intracellular ROS. We found that the PI3K regulatory subunits are not necessary for formation of the early phagosome cup or for production of extracellular ROS. However, we show that the loss of p85α leads to enhanced intracellular ROS, which also contributed to improved killing of methicillin-susceptible *S. aureus* (MSSA) and methicillin-resistant *S. aureus* (MRSA).

Our work provides a novel target in the regulation of enhancing neutrophil intracellular ROS, which has been shown to cooperate with anti-microbial agents to increase bacterial killing [22, 23]. This is an improvement over indiscriminately increasing global ROS production, which could lead to inflammation-induced tissue injury. Using intracellular ROS to augment anti-microbial therapies may provide a novel strategy in the treatment of antibiotic-resistant pathogens.

## RESULTS AND DISCUSSION

### Loss of regulatory subunits p85α, p55α, and p50α decreases extracellular ROS production, but increases intracellular ROS production

**Figure 1:**
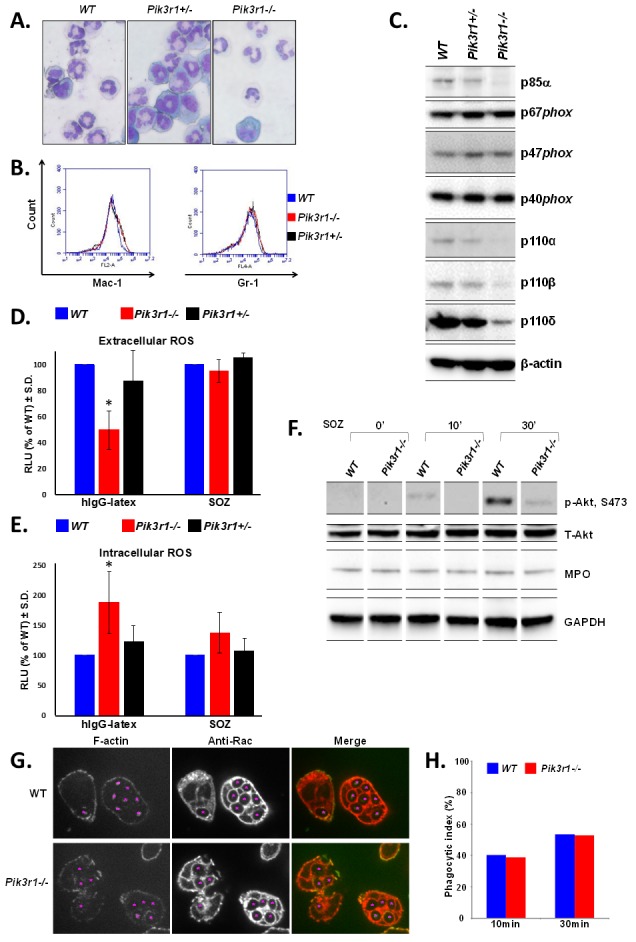
Characterization of WT, *Pik3r1+/−*, and *Pik3r1−/−* fetal liver-derived neutrophils Differentiation of fetal liver cells taken from WT, *Pik3r1^+/−^*, and *Pik3r1^−/−^* embryos was assessed based on **A**. morphology (images taken with 40X objective) and **B**. Mac-1/Gr-1 staining; **C.** Protein expression of PI3K catalytic subunits and NADPH oxidase subunits as measured by immunoblot; **D.** Extracellular and **E.** intracellular ROS production was measured in WT, *Pik3r1^+/−^*, and *Pik3r1^−/−^* fetal liver-derived neutrophils stimulated with hIgG-latex (FcγR simulation) and SOZ (CR stimulation), n=10, *p<0.0001 comparing extracellular ROS production from *Pik3r1^−/−^* to WT in response to hIgG-latex, and n=10, p=0.0009 comparing intracellular ROS production from *Pik3r1^−/−^* to WT in response to hIgG-latex, statistical analyses performed by two-tailed, one-sample Student's t-test; **F.** Immunoblotting for phospho-Akt and MPO in WT and *Pik3r1^−/−^* fetal liver-derived neutrophils, stimulated for 0min, 10min, and 30min with SOZ; **G.** WT and *Pik3r1^−/−^* fetal liver-derived neutrophils were stimulated with SOZ and immunostained with anti-F-actin and anti-Rac to visualize phagosomes and quantitate phagocytic index, images taken with 100× objective; **H.** 10min and 30min after SOZ stimulation, phagocytic index (PI) was calculated as PI = (% of phagocytic cells containing ≥ 1 particle) × (mean number of particles/phagocytic cell containing particles).

As global knockout of *Pik3r1^−/−^* leads to perinatal lethality [24], timed matings were performed between *Pik3r1^+/−^* mice for the isolation of fetal liver-derived hematopoietic cells at 14.5 days post-conception. WT (*Pik3r1^+/+^*), *Pik3r1^+/−^*, and *Pik3r1^−/−^* (lacking expression of p85α, p55α, and p50α) [24, 25] fetal liver mononuclear cells were differentiated *in vitro* to neutrophils [20], and each population demonstrated similar differentiation as assessed by morphology and Mac-1/Gr-1 staining (Figure 1A and 1B). Consistent with previous studies reporting increased PI3K catalytic subunit degradation upon loss of the stabilizing regulatory subunits [24, 26], p110α, p110β, and p110δ protein expression was substantially reduced in the *Pik3r1^−/−^* neutrophils (Figure 1C). Notably, however, the levels of the NADPH oxidase subunits (p67*^phox^*, p47*^phox^*, and p40*^phox^*) were unchanged (Figure 1C).

To examine the function of the PI3K regulatory subunits in neutrophil ROS production, we examined both extra- and intracellular ROS production in response to various stimuli. *Pik3r1^−/−^* neutrophils had significantly reduced FcγR (hIgG-latex)-stimulated extracellular ROS (50% of WT, Figure 1D), but similar amounts of CR (SOZ)-stimulated extracellular ROS (Figure 1D). Together with the lower expression of the PI3K catalytic subunits, this finding supports the notion that PI3K activity is required for extracellular ROS mediated by FcγR stimulation, and is consistent with our previous findings [20]. In contrast to that observed with extracellular ROS, loss of p85α/p55α/p50α led to significantly increased FcγR- and a trend of enhanced CR-stimulated intracellular ROS production (Figure 1E). Both extra- and intracellular ROS production from heterozygous *Pik3r1^+/−^* neutrophils was similar to that of WT.

Immunoblots confirmed that activated Akt was reduced in *Pik3r1^−/−^* neutrophils; however, myeloperoxidase (MPO) levels were equal in WT and *Pik3r1^−/−^* cells (Figure 1F), demonstrating that the increased intracellular ROS production in *Pik3r1^−/−^* neutrophils is due to a regulatory effect of p85α/p55α/p50α, rather than due to reduced MPO expression and diminished ROS consumption. Furthermore, by immunostaining with anti-F-actin and anti-Rac to visualize the phagosomes at 10min and 30min post-SOZ stimulation, we found a similar phagocytic index in WT and *Pik3r1^−/−^* neutrophils (Figure 1G and 1H), indicating that increased FcγR- and CR-stimulated intracellular ROS levels in *Pik3r1^−/−^* neutrophils is not merely due to enhanced phagocytosis, but to a regulatory role of p85α/p55α/p50α on NADPH oxidase activity.

### Re-introduction of p85α corrects the levels of extra- and intracellular ROS production in *Pik3r1^−/−^* neutrophils

**Figure 2:**
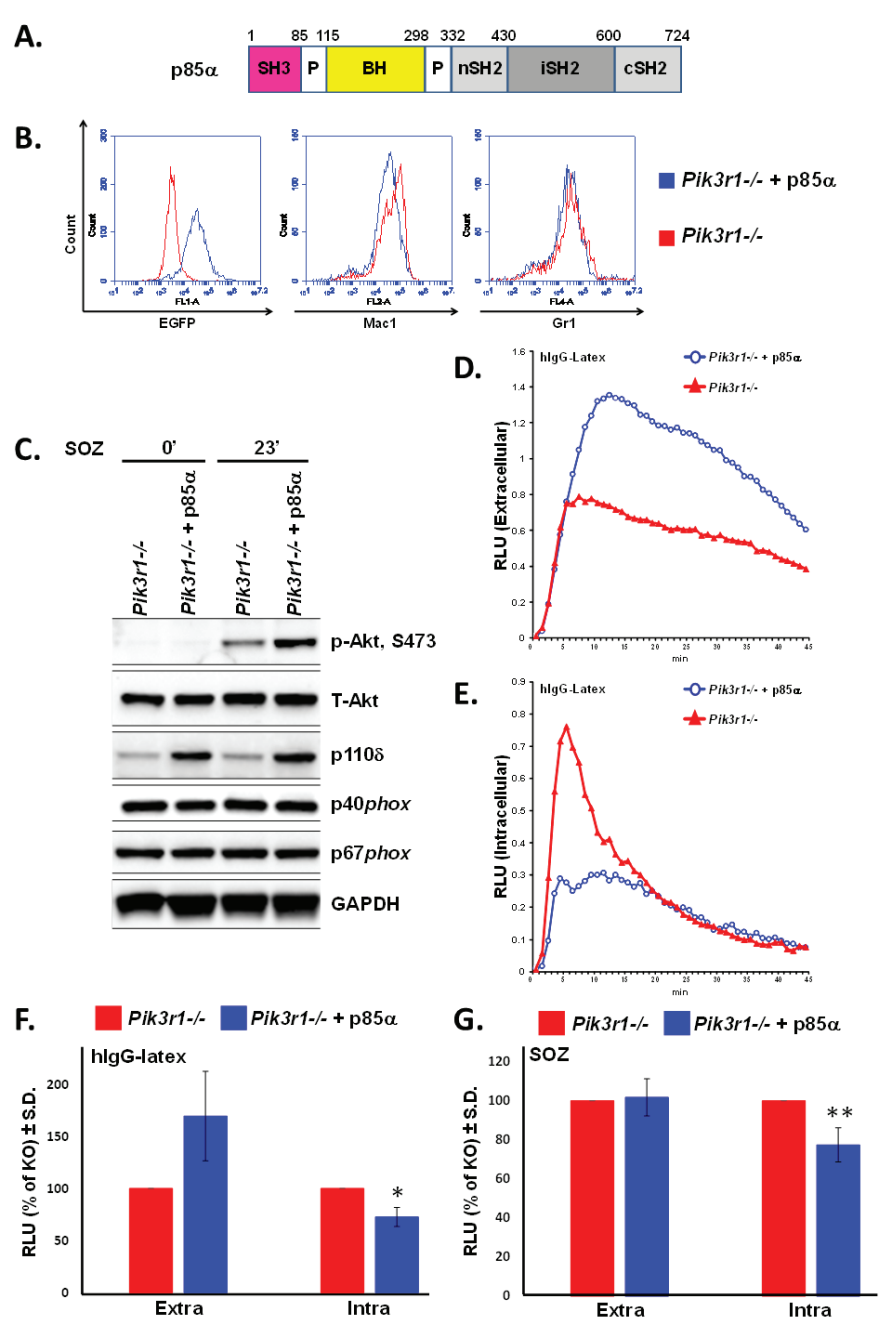
Re-introduction of p85α in *Pik3r1^−/−^* fetal liver-derived neutrophils normalizes hIgG-latex-stimulated extra- and intracellular ROS **A.** Domain structure of p85α construct, BH: Bcr homology domain; **B.** Representative flow cytometry of fetal liver-derived neutrophils upon transduction with p85α (either in tandem with EGFP or tagged with YFP); **C.** Immunoblotting for Akt, PI3K catalytic subunit p110δ, and NADPH oxidase subunits in *Pik3r1^−/−^* and *Pik3r1^−/−^* + p85α neutrophils, stimulated for 0 and 23min with SOZ; **D.** Representative extracellular and **E.** representative intracellular ROS production measured in *Pik3r1^−/−^* and *Pik3r1^−/−^* + p85α neutrophils stimulated with hIgG-latex; **F.** Quantitative assessment of hIgG-latex-stimulated extra- and intracellular ROS production, *n* = 6, *p = 0.04 comparing intracellular ROS production from *Pik3r1^−/−^* to *Pik3r1^−/−^* + p85α; **G.** Quantitative assessment of SOZ-stimulated extra- and intracellular ROS production, *n* = 7, **p = 0.05 comparing intracellular ROS production from *Pik3r1^−/−^* to *Pik3r1^−/−^* + p85α; statistical analyses performed by two-tailed, one-sample Student's *t*-test.

We next examined the effect of re-introducing p85α (Figure 2A) on extra- and intracellular ROS production from *Pik3r1^−/−^* fetal liver-derived neutrophils [27]. We found comparable neutrophil differentiation (Mac-1, Gr-1) between *Pik3r1^−/−^* neutrophils and *Pik3r1^−/−^* neutrophils upon re-introduction of p85α (Figure 2B). Protein levels of PI3K catalytic subunit p110δ were increased upon re-introduction of p85α, and concordantly, Akt phosphorylation was normalized (Figure 2C). Moreover, p85α restored hIgG-latex-stimulated extracellular ROS levels and inhibited hIgG-latex-stimulated intracellular ROS compared to *Pik3r1^−/−^* neutrophils (Figures 2D, 2E, and 2F). Consistent with a dispensable role of p85 α, p55α, and p50α on SOZ-stimulated extracellular ROS (Figure 1D), re-introduction of p85α did not alter SOZ-stimulated extracellular ROS production from *Pik3r1^−/−^* neutrophils (Figure 2G); however, SOZ-stimulated intracellular ROS was inhibited by re-introduction of p85α. These findings demonstrate that of the PI3K regulatory subunits, p85α uniquely is able to negatively regulate hIgG-latex- and SOZ-stimulated intracellular ROS production. Furthermore, as the C-terminal portion of p85α (nSH2, iSH2, and cSH2 domains, the shared domains between the p85α, p55α, and p50α regulatory proteins) is critical for promoting PI3K activity, these findings suggest that the N-terminus of p85α (SH3 and BH domains) functions to negatively regulate intracellular ROS.

### Elevated intracellular ROS in *Pik3r1^−/−^* neutrophils enhances bacterial killing

**Figure 3:**
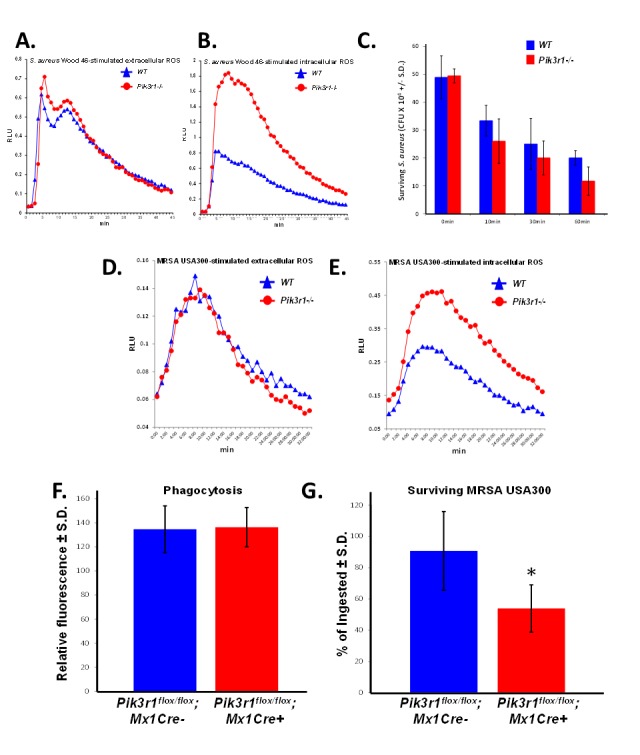
*Pik3r1^−/−^* neutrophils demonstrate superior killing of MSSA (Wood 46) and MRSA (USA300) **A.** Extracellular and **B.** intracellular ROS production from WT and *Pik3r1^−/−^* fetal liver-derived neutrophils in response to serum-opsonized MSSA (Wood 46), representative of 2 independent experiments; **C.** WT and *Pik3r1^−/−^* fetal liver-derived neutrophil killing of MSSA (Wood 46) was measured by counting the number of surviving bacteria after 0, 10, 30, and 60min incubation, *n* = 3, *p* = 0.09 comparing *Pik3r1^−/−^* to WT at 60min, statistical analysis performed by unpaired, two-tailed Student's t-test; **D.** Extracellular and **E.** intracellular ROS production was measured in WT and *Pik3r1^−/−^* neutrophils stimulated with serum-opsonized MRSA (USA300), experiment performed on one occasion. **F.** Phagocytosis and **G.** killing of MRSA (USA300) by *Pik3r1^flox/flox^*; *Mx1Cre^−^* and *Pik3r1^flox/flox^*; *Mx1Cre^+^* bone marrow neutrophils was measured by fluorescence remaining inside cells after washing and quenching extracellular fluorescence, *n* = 30, *p < 0.001 comparing *Pik3r1^flox/flox^*; *Mx1Cre^−^* to *Pik3r1^flox/flox^*; *Mx1Cre^+^*, statistical analysis by unpaired, two-tailed Student's *t*-test, experiment conducted on two independent occasions.

Based on our observation that *Pik3r1^−/−^* fetal liver-derived neutrophils have increased intracellular ROS production in response to Fcγ receptor and CR stimulation, we predicted that *Pik3r1^−/−^* neutrophils would demonstrate enhanced *S. aureus*-stimulated ROS production and enhanced bacterial killing compared to WT neutrophils. Consistent with our hypothesis, *Pik3r1^−/−^* fetal liver-derived neutrophils produce more intracellular ROS in response to serum-opsonized MSSA (Wood 46), while the extracellular ROS levels were not affected (Figures 3A and 3B).

To determine the antimicrobial function of *Pik3r1^−/−^* neutrophils, we performed a bacterial killing assay. WT and *Pik3r1^−/−^* fetal liver-derived neutrophils were incubated with serum-opsonized methicillin-sensitive S. aureus (MSSA) (Wood 46) over 60 min, and at various time points, neutrophil samples were quenched in ice-cold LB-saponin, sonicated to liberate ingested bacteria, and surviving bacteria were enumerated by plating on LB-agar. Surviving MSSA was reduced when incubated with *Pik3r1^−/−^* neutrophils compared to WT neutrophils at all time points, with a trend toward statistical significance at 60 minutes (Figure 3C).

Given the promising results using fetal liver-derived neutrophils and Wood 46 MSSA, we turned our attention to a more relevant pathogen, MRSA. Hypervirulent MRSA, such as LAC MRSA USA300 strain, has become an important public health problem due to the sheer number of infections and widespread antibiotic resistance [3]. Consistent with results observed using MSSA, we observed enhanced intracellular ROS in fetal liver-derived *Pik3r1^−/−^* neutrophils compared to WT neutrophils in response to serum-opsonized MRSA (USA300), and no difference in extracellular ROS production (Figures 3D and 3E). We next conducted bacterial killing assays using a more physiologic source of neutrophils directly isolated from the bone marrow using a percoll gradient. Since global knockout of *Pik3r1* induces perinatal lethality, we utilized a murine model bearing a conditionally targeted *Pik3r1* allele (*Pik3r1*^flox/flox^) crossed with Mx1-Cre. *Pik3r1*^flox/flox^;Mx1Cre^+^ and *Pik3r1*^flox/flox^;Mx1Cre^−^ littermate controls were treated with polyI:polyC to induce recombination of the *Pik3r1* allele. Animals were permitted to recover from polyI:polyC treatment for at least 12 weeks prior to isolation of bone marrow neutrophils. Phenotypically and morphologically, bone marrow neutrophils isolated directly from the *Pik3r1*^flox/flox^;Mx1Cre^+^ and *Pik3r1*^flox/flox^;Mx1Cre^−^ bone marrow were similar (data not shown). To control for phagocytosis, neutrophils were incubated with GFP-expressing MRSA for 2 hours, followed by quenching of extracellular GFP using trypan blue. To measure bacterial killing, a second plate was incubated for an additional 2 hours and then quenched with trypan blue. Following quenching, intracellular GFP levels were read immediately on a fluorometer to measure phagocytosis and bacterial killing, respectively. GFP-expressing MRSA were phagocytized equally (Figure 3F); however, significantly reduced GFP levels, previously shown to correlate with bacterial survival [28], was observed in *Pik3r1*^flox/flox^;Mx1Cre^+^ neutrophils compared to *Pik3r1*^flox/flox^;Mx1Cre^−^ neutrophils (Figure 3G). Collectively, these findings suggest that increased intracellular ROS production may provide enhanced bacterial killing, in particular of MSSA and MRSA.

Our findings demonstrate that genetic disruption of *Pi3kr1* differentially regulates NADPH oxidase activity on the plasma membrane (extracellular ROS production) v. the phagosome membrane (intracellular ROS production). These novel findings are consistent with other studies demonstrating that the regulation of NADPH oxidase activity differs between the plasma and phagosome membranes, and highlights the varied environments of these two compartments. An example relevant to the current work is the Class III PI3K product, PI3P, which is a strong positive regulator of intracellular ROS production but plays no role in extracellular ROS production. A key molecule increasing Class III PI3K activity and increased PI3P production on the phagosome is the Rab GTPase, Rab5. Down-regulation of Rab5 reduces the capacity of *S. aureus*-containing phagosomes to fuse with endocytic organelles resulting in poorer bacterial killing [29]. Notably, p85α is known to interact with Rab5 and to bear GTPase Activating Protein (GAP) activity towards Rab5-GTP, which is localized to the p85α BH domain (Figure 2A). These considerations suggest that a feasible mechanism underlying the negative regulatory effect of p85 α on intracellular ROS may be downregulating Rab5a-GTP levels *via* its GAP function [30, 31], resulting in reduced Class III PI3K-derived PI3P. Thus, while our current work cannot exclude the possibility that p85α negatively regulates intracellular ROS production in an indirect manner due to altered expression of the Class IA PI3K catalytic subunits, a thought-provoking consideration is that p85 α functions in a Class IA PI3K catalytic subunit-independent manner to regulate NADPH oxidase activity on the phagosome membrane.

Collectively, our findings show that neutrophils lacking the PI3K regulatory subunit p85α produce significantly more intracellular ROS without affecting phagocytosis. Furthermore, this correlates to significantly increased killing of both MSSA and MRSA. These results suggest a new strategy for combating the growing threat of resistant microorganisms.

## MATERIALS AND METHODS

### Preparation of particulate stimuli

Human IgG-opsonized latex beads (1.98 μm or 2.94μm), serum opsonized-zymosan A particles (Sigma, St. Louis, MO, USA), and serum-opsonized MSSA (Wood 46) were prepared as previously described [32–34]. MRSA USA300 (LAC) expressing superfolded GFP was grown overnight at 37°C with shaking, OD600 was measured to identify mid-logarithmic growth, and bacteria were washed with PBS before adding to PMNs [35]. Final concentrations were 5:1 IgG-beads/PMNs; 400 μg/ml SOZ; 40:1 *S. aureus*/PMNs; 60 μg/ml hyphae; 50:1 MRSA/PMNs.

### Western blots

Cell lysates were prepared from murine neutrophils using 1% Triton X-100, 15 μg lysate was subjected to SDS-PAGE separation and immunoblotting using a nitrocellulose membrane (Thermo Scientific). Blots were blocked with 5% BSA and then incubated overnight with anti-p85α, -p110δ (Santa Cruz);anti-p67phox, -p47phox, -p40phox (Millipore); anti-p110a, -p110b, -phospho-Akt (Ser473), or -MPO (Cell Signaling). Blots were then incubated with HRP-conjugated secondary antibody and signal was visualized with ECL detection (Pierce).

### Confocal microscopy

SOZ-induced phagocytosis in WT and *Pik3r1^−/−^* fetal liver-derived neutrophils was imaged using a spinning-disk (CSU10) confocal system mounted on a Nikon TE-2000U inverted microscope with an Ixon air-cooled EMCCD camera (Andor Technology, South Windsor, CT) and a Nikon Plan Apo 100X 1.4 N.A. objective as described previously [16, 20, 21]. Images were analyzed with Metamorph software (Universal Imaging; Downington, PA).

### Animal husbandry

Mice were housed and bred in accordance with the Institutional Animal Care and Use Committee of the Indiana University School of Medicine. *Pik3r1^+/−^* mice [24, 25] were subjected to timed matings, dams were euthanized at day 14 post-conception, and fetal livers were harvested for generation of *Pik3r1^+/+^, Pik3r1^+/−^*, and *Pik3r1^−/−^* neutrophils. *Pik3r1^flox/flox^* mice [36] were crossed with Mx1-Cre^+^ mice to produce *Pik3r1^flox/flox^*; Mx1-Cre^+^ mice. Cre expression was induced by 3 intraperitoneal injections of polyI:polyC (300ug), and recombination of *Pik3r1* was confirmed by genotyping.

### Neutrophil differentiation

Fetal liver cells were collected from embryos at day 14 of gestation, genotyped, and differentiated into neutrophils in α-minimum essential medium with 20% heat-inactivated FCS, 1% penicillin/streptomycin, 50ng/ml G-CSF and 50units/ml IL-3. Every 2 days, cells were counted and replated at a concentration of 0.5×10^6^/ml in fresh differentiation medium. Activity was analyzed on days 6 and 7 of differentiation [20].

### Reintroduction of p85α

p85α cDNA was cloned upstream of an internal entry site (IRES) and the enhanced green fluorescence protein (EGFP) in the bicistronic retroviral plasmid, pMIEG3 [37]. Alternatively, p85α was tagged on the C-terminal end with yellow fluorescent protein (YFP) and cloned into the retroviral plasmid, pMSCV (Clontech). Ecotropic retrovirus containing the vectors was used to transduce *Pik3r1^−/−^* fetal liver cells, cells were sorted for EGFP or YFP positivity, and differentiated into neutrophils. Data using both constructs are combined for statistical analyses, as the YFP tag did not alter the function of p85α (data not shown).

### ROS detected by chemiluminescence in intact cells

Oxidant production was measured by chemiluminescence using 2×10^5^ neutrophils during synchronized phagocytosis of particulate stimuli [20, 21, 38, 39]. Extracellular ROS was measured using 20μM isoluminol with 20U/ml horseradish peroxidase (HRP), and intracellular ROS was measured using 20μM luminol with 20U/ml HRP and 10μg/ml superoxide dismutase (SOD) [20, 21, 32]. An Lmax microplate luminometer (Molecular Devices, Sunnyvale, CA, USA) was used to record luminescence as previously described [20, 21].

### Methicillin-susceptible *S. aureus* (Wood 46) killing

6×10^6^ WT or *Pik3r1*^−/−^ fetal liver-derived neutrophils were incubated with 1.5×10^6^ serum-opsonized MSSA (strain Wood 46) in 300 μl PBS for 60 min. Samples were added to ice-cold Difco nutrient broth (BD, Franklin Lakes, NJ, USA) with 10% saponin and sonicated to liberate ingested bacteria [32]. Surviving bacteria were enumerated by plating on Columbia agar (Sigma, St. Louis, MO, USA).

### Neutrophil isolation from bone marrow and MRSA (USA300 LAC) killing

Mice were sacrificed, bone marrow cells were collected from the pelvis, femur, and tibia, and neutrophils were isolated using a 62% and 55% percoll gradient. Neutrophils were washed in Hanks' Balanced Salt solution (Sigma, St. Louis, MO, USA), resuspended in Iscove's Modified Dulbecco's Medium (Life Technologies, Carlsbad, CA, USA), and plated at 2×10^5^ cells per well into a 96well plate coated with celltak (Corning, Corning, NY, USA). MRSA (USA300 strain LAC) was incubated with neutrophils for 2h at 37°C, and then plates were washed with warm PBS. For phagocytosis analysis, 50uL of 500mg/mL trypan blue was added immediately to PBS-washed plates to quench extracellular fluorescence. For bacterial survival analysis, PBS-washed plates were incubated for an additional 2h at 37°C followed by the addition of 50uL of 500mg/mL trypan blue. For both phagocytosis and bacterial killing analyses, plates were read on a fluorometer to measure intracellular GFP fluorescence promptly following the addition of trypan blue. Fluorescence intensity correlates to bacterial survival based on the previously defined correlation of HOCl-bleaching of superfolded GFP to bacterial viability.

## Abbreviations

CGD: chronic granulomatous disease
CR: complement receptor
FcγR: Fcγ receptor
hIgG: Human IgG-opsonized latex beads
HRP: horseradish peroxidase
MPO: myeloperoxidase
MRSA: methicillin-resistant S. aureus
MSSA: methicillin-susceptible S. aureus
PI3K: phosphoinositide 3-kinase
PMN: polymorphonuclear neutrophil
ROS: reactive oxygen species
S. aureus: Staphylococcus aureus
SOD: superoxide dismutase
SOZ: serum opsonized zymosan
